# Timing and periodicity of Phanerozoic marine biodiversity and environmental change

**DOI:** 10.1038/s41598-019-42538-7

**Published:** 2019-04-16

**Authors:** Gareth G. Roberts, Philip D. Mannion

**Affiliations:** 1 Department of Earth Science and Engineering, Imperial College London, South Kensington Campus, London, SW7 2AZ UK; 2 Present Address: Department of Earth Sciences, University College London, London, WC1E 6BT UK

## Abstract

We examine how the history of Phanerozoic marine biodiversity relates to environmental change. Our focus is on North America, which has a relatively densely sampled history. By transforming time series into the time-frequency domain using wavelets, histories of biodiversity are shown to be similar to sea level, temperature and oceanic chemistry at multiple timescales. Fluctuations in sea level play an important role in driving Phanerozoic biodiversity at timescales >50 Myr, and during finite intervals at shorter periods. Subsampled and transformed marine genera time series reinforce the idea that Permian-Triassic, Triassic-Jurassic, and Cretaceous-Paleogene mass extinctions were geologically rapid, whereas the Ordovician-Silurian and Late Devonian ‘events’ were longer lived. High cross wavelet power indicates that biodiversity is most similar to environmental variables (sea level, plate fragmentation, *δ*^18^O, *δ*^13^C, *δ*^34^S and ^87^Sr/^86^Sr) at periods >200 Myr, when they are broadly in phase (i.e. no time lag). They are also similar at shorter periods for finite durations of time (e.g. during some mass extinctions). These results suggest that long timescale processes (e.g. plate kinematics) are the primary drivers of biodiversity, whilst processes with significant variability at shorter periods (e.g. glacio-eustasy, continental uplift and erosion, volcanism, asteroid impact) play a moderating role. Wavelet transforms are a useful approach for isolating information about times and frequencies of biological activity and commonalities with environmental variables.

## Introduction

Biodiversity is regulated by some combination of biotic, geologic and climatic processes^1–9^. For example, the diversity dynamics of marine species are a complex response to a suite of variables including ecology, sea level, continental configuration, and the temperature and chemistry of oceans. Whereas biotic factors are often considered to have shaped diversity over short time intervals and primarily locally (the Red Queen model), extrinsic factors are generally perceived to have exerted their influence over longer time periods and much wider geographic ranges (the Court Jester model^4,10^). Although a number of studies indicate a more complex interplay of biotic and abiotic variables than this oversimplified dichotomy would suggest^5,11–14^, extrinsic factors clearly play a key role in driving macroevolution^8^. Disentangling how geological and environmental variables and biodiversity interact thus has important implications for understanding the evolutionary history of life on Earth, including the role of mass extinctions^6,8,12^.

**Figure 1:**
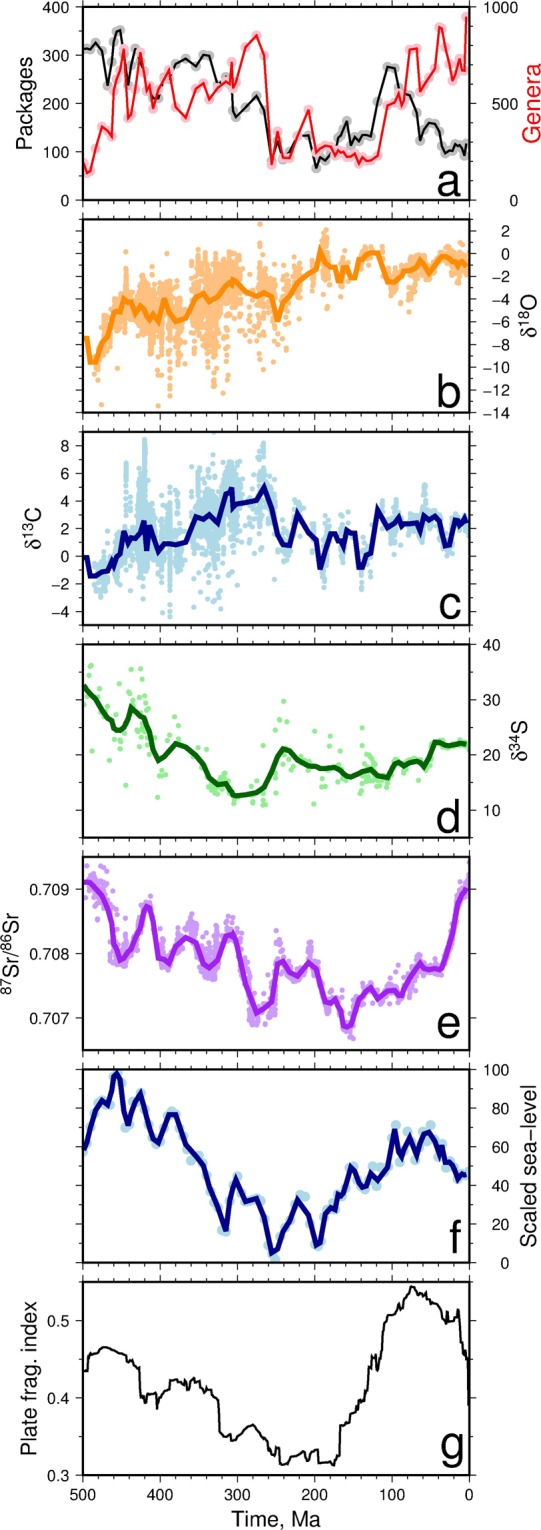
(**a**) Red = total number of marine genera from North American fossil occurrences; black = total number of marine sedimentary packages in North America. (**b**–**f**) Environmental variables from^6^. (**b**–**e**) Isotopic ratios from marine carbonates. *δ*^18^*O* (orange) and *δ*^13^*C* (blue) are from low latitudes. Solid lines = average for time bins in panel (a). (**f**) Circles = Continental flooding; solid line = moving average binned for time series shown in panel a^6^. (**g**) Plate fragmentation index^9^.

A critical step towards understanding how environmental variables affect biodiversity is the identification of timescales and times at which their signals are generated. Another is quantification of similarities and disparities between signals. In this study we focus on the biodiversity of North American marine organisms, which have a rich, densely sampled, and continuous fossil record throughout the Phanerozoic (the last 541 million years^15–19^), and have been the subject of the vast majority of studies examining the deep time drivers of biodiversity^5–7,9,20,21^. We focus on analysing the well known and widely used time series generated by *Hannisdal & Peters*^6^ from the Paleobiology Database (PBDB). We also examine the implications of using a more up-to-date dataset. Visual inspection of Fig. 1 shows that the history of some environmental variables is similar to that of marine genera on long (>100 Myr) timescales. For example, temporal fluctuations in the number of genera have a broad peak-trough-peak shape during the last 500 Myr, which is similar to the history of sea level changes (cf. Fig. 1a,f). Taken at face value, this similarity is suggestive of simple deterministic behaviour, i.e. high sea level generates more habitable space for marine organisms to diversify.

It would be useful to know the timescales at which this apparently deterministic behaviour applies and if similarities exist between this and other environmental variables. It would also be useful to know if biodiversity experiences hysteresis, i.e. is there a lag between a change in an environmental variable and biodiversity? One way to approach this problem is to transform time series into the frequency domain. Directly relevant to our study is the time series analysis of paleoclimate proxies, isotopic ratios in marine carbonates, and the comparison of large igneous provinces to marine biodiversity and ocean chemistry^6,22,23^. *Hannisdal & Peters*^6^ developed a probabilistic framework to assess whether time series were coupled and if certain signals (e.g. sea level) could be considered drivers of others (e.g. genera). This approach uses whole time series to assess ‘information transfer’ between signals, which is a significant advance over simple first-difference approaches. *Prokoph & El Bilali*^22^ and *Prokoph et al*.^24^ used a wavelet transform approach to solve for power as a function of time and frequency and identified similarities between time series and phase differences in the time-frequency domain using cross wavelet transforms (see also^25^). We build on that work to compare biodiversity to a more complete suite of time series related to reputed driving processes (e.g. isotopic proxies, sea level, plate fragmentation). A focus of this study is to understand how, or if, changes in putative driving processes correlate with, and perhaps drive, biodiversity through time. We use continuous wavelet transforms to address this issue. A benefit to using wavelet transforms, rather than, say, Fourier transforms or probabilistic approaches, is that we can estimate the power of signals as a function of time and frequency. Wavelet transforms have been used in a variety of fields to identify where spectral power of signals resides (e.g. geophysics, oceanography, cardiology, climatology, economics).

In essence, a time series, i.e. signals with amplitudes that vary as a function of time, *x*(*n*), are transformed into ‘maps’ of amplitude as a function of time and frequency, *x*(*n*, *ω*). It is then straightforward to quantify signal power (squared amplitudes) as a function of time and frequency, which helps to identify, for example, finite duration periodicities in time series. In this study, transformation is performed using a nonorthogonal continuous wavelets approach, which is useful for time series with amplitudes that are expected to vary continuously and smoothly (e.g.^26^). The transformed time series include number of marine genera and sedimentary rock packages in North America, isotopic ratios from marine carbonates at low latitudes (*δ*^18^O, *δ*^13^C, *δ*^34^S, ^87^Sr/^86^Sr) and estimates of continental flooding and plate fragmentation^6,24^ (Fig. 1). We compare these power spectra to spectra calculated using a diversity estimate of North American marine genera. Time series are compared using cross wavelet transforms from which we extract information about the phase difference of the time series, which contains information about biodiversity hysteresis. Finally, we use our results to suggest causal links between environmental variables and biodiversity.

## Methodology

### Wavelet transforms

The continuous wavelet transform of discrete measurements (e.g. *x* at times *n*) is the convolution of *x*_*n*_ with a scaled and translated mother wavelet function, *ψ*. A Morlet wavelet function was used to transform the time series in this study (see^26^). The wavelet transform *W*_*x*_ can be written in discrete notation as

1 $${W}_{x}(s)=\sum _{n^{\prime} \mathrm{=0}}^{N-1}{x}_{n^{\prime} }\psi [\frac{(n^{\prime} -n)\delta t}{s}].$$

The mother wavelet is stretched in time by scale, *s*, and translated along the time series by *n*′. This transform can be used to show how amplitudes vary in time-frequency space for *N* data points. Frequencies are obtained from scales using equivalent Fourier periods (see^26^). An inverse wavelet transform can be performed by summing the wavelet transform over all scales. The wavelet power spectrum is given by

2 $$\varphi (s,x^{\prime} )=|{W}_{x}(s{)|}^{2},$$

and the time-average power spectrum is

3 $$\bar{\varphi }(s)=\frac{1}{N}\sum _{x\mathrm{=0}}^{N-1}|{W}_{x}(s{)|}^{2}\mathrm{.}$$

The time-average power spectrum depends on scale. We rectified this spectral bias by calculating *ϕ*_*r*_ = *ϕ*(*s*)|*s*^−1^|^27^.

### Time series

We used *Hannisdal & Peters*^6^’s genera time series in our analyses (solid lines in Fig. 1). Their time series was constructed from marine genera extracted from the PBDB. The taxa used to generate the time series included *Cetacea, Pinnipedimorpha, Pinnipedia, Otariidae, Phocoidea, Phocidae, Otarioidea, Odobenidae, Enhydra, Sirenia, Chelonioidea, Cheloniidae, Dermochelyidae, Protostegidae, Toxochelyidae, Thalassemyidae, Plesiosauria, Sauropterygia, Mosasauridae, Ichthyosauria, Thalattosuchia, Enaliosauria, Nothosauroidea, Placodontia, Thalattosauria, Brachiopoda, Echinodermata, Cnidaria, Bryozoa, Porifera, Archaeocyatha, Hemichordata, Annelida, Priapulida, Granuloreticulosea, Actinopoda, Haptophyta, Bacillariophyta, Rhodophyta, Chlorophyta, Cyanobacteria, Rhizopodea, Bacillariophyceae, Pachycormiformes, Conodontophorida, Pleuronectiformes, Cephalopoda, Polyplacophora, Calpionellida, Aplacophora, Helcionelloida, Monoplacophora, Rostroconchia, Scaphopoda, Conodonta, Tunicata, Trilobita, Merostomata, Mollusca, Elasmobranchii*. We also transformed the standardized subsampled marine organisms time series from *Hannisdal & Peters*^6^. This time series was produced using the shareholder quorum subsampling (SQS) approach that aims to extract a ‘fixed proportion of the species pool’ from, for example, numbers of observed genera^28^.

To investigate the source of power in the marine animal time series we also transformed the number of Phanerozoic rock packages, plate fragmentation, sea level and stable isotopic time series. The isotopic time series transformed were: *δ*^18^O, *δ*^13^C; *δ*^34^S, ^87^Sr/^86^Sr. Where

4 $$\delta {}^{18}{\rm{O}}=(\frac{{}^{18}{\rm{O}}/{}^{16}{\rm{O}}_{sample}}{{}^{18}{\rm{O}}/{}^{16}{\rm{O}}_{standard}}-1)\times \mathrm{1000,}$$

5 $$\delta {}^{13}{\rm{C}}=(\frac{{}^{13}{\rm{C}}/{}^{12}{\rm{C}}_{sample}}{{}^{13}{\rm{C}}/{}^{12}{\rm{C}}_{standard}}-1)\times \mathrm{1000,}$$

6 $$\delta {}^{34}{\rm{S}}=(\frac{{}^{34}{\rm{S}}/{}^{32}{\rm{S}}_{sample}}{{}^{34}{\rm{S}}/{}^{32}{\rm{S}}_{standard}}-1)\times 1000.$$

Sea level was extracted from^29^ who produced a eustatic sea-level time series based on *Haq et al*.^30^ and *Haq & Schutter*^31^’s global synthesis (Fig. 1f). Prior to transformation all time series were downsampled, such that *δt* = 3 Ma, so that they could be compared to the relatively sparsely sampled genera time series. We acknowledge that this process reduces high frequency content of isotopic time series and that we are restricted to discussing periods ≳3 Myr. The time series transformed are averages for the time bins shown in Fig. 1a (see^6^). The data were mirrored to reduce the impact of edge effects on calculated spectra. We normalised the mirrored data to zero mean and unit variance^6^. The time-frequency content of signals were compared by calculating their cross wavelet spectra, which is in somewhat analogous to coherence calculated using classical Fourier techniques^32^.

Since *Hannisdal & Peters*^6^ paper was published, the PBDB has been updated and ages have been standardised using the 2012 Geological Time Scale. To investigate the implications of these changes for our results we transformed an updated time series generated using the same taxa as *Hannisdal & Peters*^6^ (see Supplementary Information). Encouragingly, calculated power spectra are similar to those calculated for *Hannisdal & Peters*^6^ raw genera and SQS time series. In general the updated time series contains more high frequency content, which is probably to be expected as more samples are included in the database. The two most prominent changes include an increase in signal amplitude (and power) between the end-Permian and end-Triassic extinction events, as well as higher amplitude, high frequency content around the Late Ordovician extinction ‘event’. Elsewhere the time series (and resultant power spectra) are similar. For the sake of consistency with previous work our subsequent analyses are applied to *Hannisdal & Peters*^6^’s SQS time series but could be applied to most paleobiological time series.

### Cross wavelet spectra

Using wavelet transforms ${W}_{n}^{X}(s)$ and ${W}_{n}^{Y}(s)$ of time series *x*_*n*_ and *y*_*n*_ we can calculate the cross wavelet spectrum, ${W}^{XY}={W}^{X}{W}^{{Y}^{\ast }}$, where * denotes complex conjugation (e.g. for an individual element in the *W*^*Y*^ matrix, *a* $\mp$ *bi* → *a* ± *bi*, where *i*^2^ = −1; see^33^). The cross wavelet power can be defined as |*W*^*XY*^| (see^26^). In practice, after converting scales to frequencies, this calculation generates a map, |*W*^*XY*^(*n*, *ω*)|, that has largest peaks where the two time series have high amplitudes at the same position in time-frequency space. We calculate time-averaged cross power spectra as

7 $$\overline{{W}^{XY}}(s)=\frac{1}{N}\sum _{n\mathrm{=0}}^{N-1}|{W}^{XY}(s\mathrm{)|.}$$

The complex argument arg(*W*^*XY*^) can be interpreted as the local phase difference between *x*_*n*_ and *y*_*n*_ in time-frequency space^26,32^. We successfully benchmarked our code against *Grinsted et al*.^32^. Supplementary Information shows cross wavelet spectra and phase calculated for a suite of synthetic examples, which help to explain the methodology and what is meant by phase differences.

## Results

### Marine genera spectra

**Figure 2:**
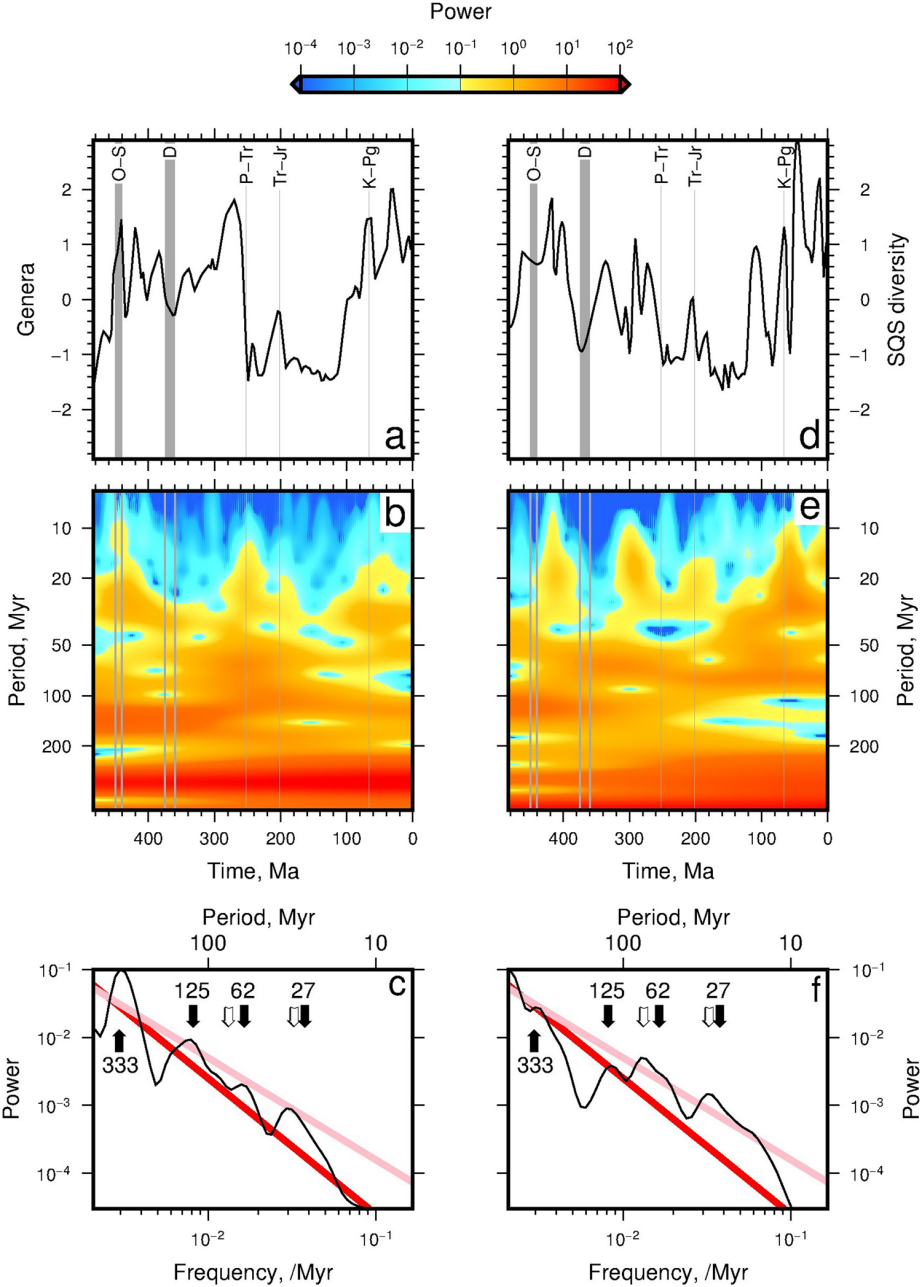
Wavelet power spectra of Genera and SQS diversity^6^. (**a**) Black line = genera time series. Gray bands = ‘big 5’ mass extinctions. (**b**) Wavelet transform power spectrum of genera. (**c**) Black = time-averaged wavelet power spectrum of genera rectified by scale. Note that highest power resides at longest periods (smallest frequencies). Red line = Brownian noise spectrum (i.e. *ϕ*_*r*_ ∝ *ω*^−2^). Pink line = pink noise spectrum (i.e. *ϕ*_*r*_ ∝ *ω*^−1.5^). Labeled black arrows = spectral peaks; annotations = periods in Myr. White arrows = spectral peaks at periods of 77 and 31 Myr. (**d**–**f**) Wavelet power spectra of SQS diversity^6^.

Figure 2 shows the power spectrum for marine genera from North American fossil occurrences^6^. The calculated power spectrum has highest power at long periods (low frequencies) and relatively short intervals of high power at shorter periods (e.g. between 450–440 Ma, 360–340 Ma). Note that high power indicates large positive or negative amplitudes. A benefit to using wavelet transforms is that signals can be easily reconstructed by summing the transform over scales (frequencies). The inverse transform reliably reproduces the original signal; error of the mean is 0.7% and 61% of the signal mean is generated at periods >98 Myr. The time-averaged power spectrum is shown in Fig. 2c.

The SQS diversity time series is broadly similar to the raw genera time series, although one notable difference is the removal of a broad peak between ∼300–240 Ma (cf. Fig. 2a,d). Figure 2e shows that highest power in the SQS diversity time series occurs at long periods, *T* > 100 Myr. Phanerozoic diversity has increased towards the present day at periods greater than 200 Myr (as demonstrated by the increased reddening shown in Fig. 2e). At periods of 100–200 Myr it has decreased throughout the Phanerozoic (i.e. bluer colours in Fig. 2e towards the present day). At shorter periods (e.g. *T* < 50 Myr) power (i.e. biodiversity) is greatest between ∼460–420 Ma and centred on ∼300 Ma, ∼200 Ma, ∼60 Ma and ∼10 Ma. Some of these time intervals coincide with mass extinction events (gray bands in Fig. 2a–b). Power between 300–180 Ma at periods of ∼30–50 Myr is lower in the SQS diversity spectrum than for the number of raw genera (cf. Fig. 2b,e). The time-averaged power spectra of the SQS time series is similar to pink noise, *ϕ*_*r*_ ∝ *ω*^−1.5^. This spectrum implies that short periods contribute more information to the SQS time series than equivalent periods in the number of raw genera time series, which is broadly similar to Brownian (red) noise, i.e. *ϕ*_*r*_ ∝ *ω*^−2^. Similar results are obtained when up-to-date PBDB time series are transformed.

Figure 2c,f show time averaged power spectra. At least four spectra peaks are observed in the raw genera and SQS time series. These peaks are centred on periods of 333 Myr, 125 Myr, 62–77 Myr and 27–31 Myr. To examine the processes that could be responsible for generating the SQS diversity spectra, time series of a suite of environmental variables were also transformed.

### Environmental variable spectra

**Figure 3:**
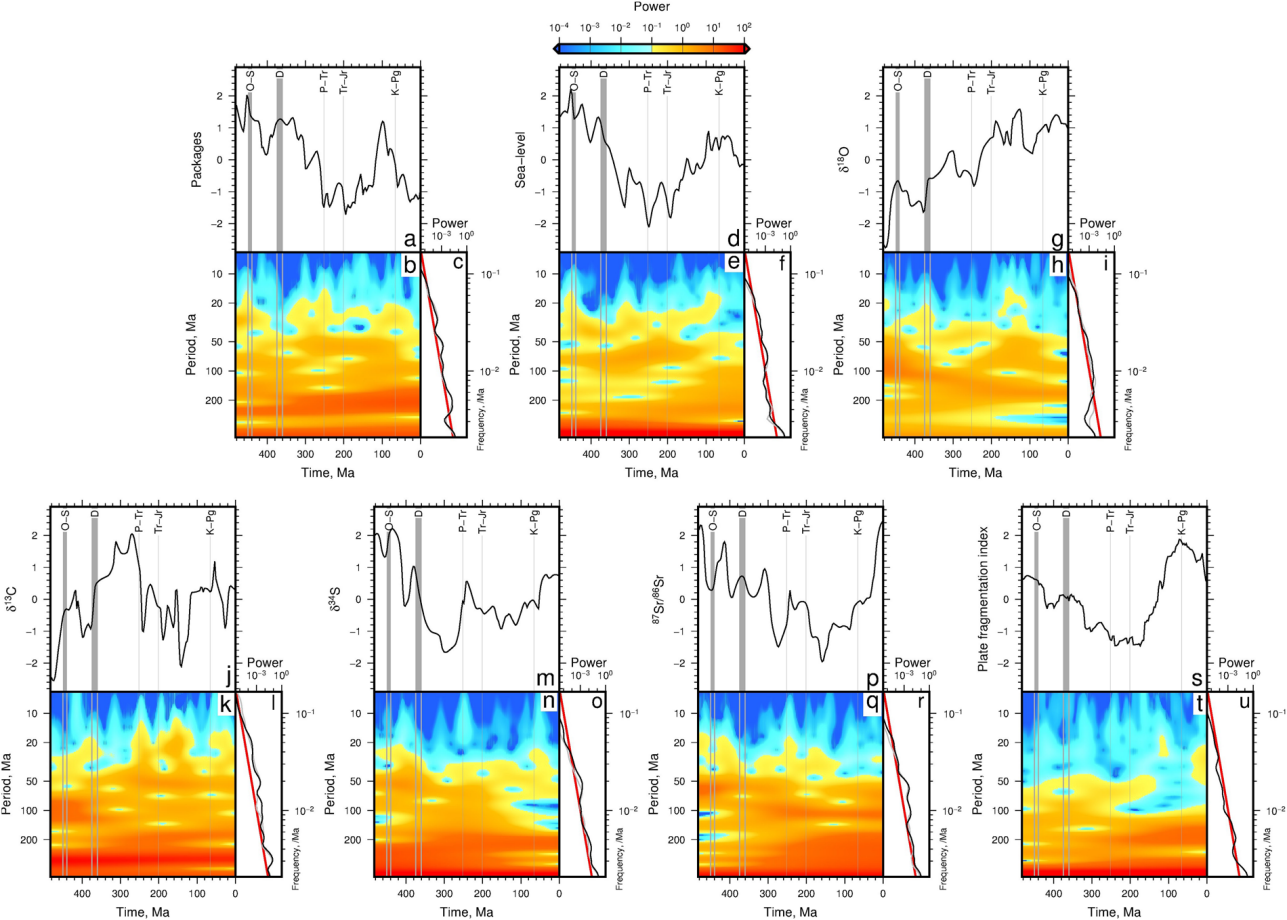
(**a**) Spectral analyses of the number of marine sedimentary packages resampled using SQS time series (see^6^). Time series were resampled to have evenly spaced data points prior to transformation (*δ*_*t*_ = 3 Ma). (**b**) Wavelet power spectrum of packages. (**c**) Black line = time-averaged power spectrum. Gray line = power spectrum for variables sampled using time-bin averages from^6^ (e.g. Fig. 1a; Supplementary Fig. 4). Red line = red noise power spectrum (i.e. power is proportional to *ω*^−2^, where *ω* is frequency). Resampling by SQS timescales makes small differences to calculated spectra. Spectra for environmental variables resampled for SQS timescale: (**d**–**f**) sea-level, (**g**–**i**) *δ*^18^O, (**j**–**l**) *δ*^13^C, (**m**–**o**) *δ*^34^S, (**p–r**) ^87^Sr/^86^Sr. (**s**–**u**) Plate fragmentation index.

Figure 3 shows wavelet power spectra for the number of marine sedimentary rock packages in North America. Highest power is at periods >200 Myr. At periods <50 Myr highest power occurs between ∼480–420 Ma, ∼340–230 Ma, and ∼90–20 Ma (see Fig. 3b). This spectra is broadly similar to the spectra of the SQS diversity and number of raw genera time series. The time-averaged spectrum is similar to Brownian noise (Fig. 3c). The wavelet spectrum of sea level is shown in Fig. 3d–f. Similar to the sedimentary rock package and SQS diversity time series, most power is concentrated at periods >200 Myr, and power is high for finite durations at periods <100 Myr.

Spectra of isotopic time series sampled using the SQS timescale are shown in Fig. 3g–r. Unsurprisingly, these power spectra are almost identical to the spectra for time series sampled using the genera time bins (cf. Fig. 3 and Supplementary Fig. 4). Nearly all of the power in the oxygen isotope (*δ*^18^O) time series, which reflects global climate change, is at periods >50 Myr (Fig. 3h). High power at periods shorter than 20 Myr is greatest between 180–80 Ma. The *δ*^13^C time series, which is a proxy for the exchange of carbon between isotopically enriched inorganic sources and depleted organic reservoirs, has greatest power at long periods (>200 Myr), as well as four intervals of high power at periods ≲20 Myr: 400–360 Ma, 280–220 Ma, 200–120 Ma, and 80–20 Ma (Fig. 3j–l). The *δ*^34^S time series, which is probably related to ocean oxygenation via microbial sulphate reduction, has less power at these short periods (Fig. 3m–o). The ^87^Sr/^86^Sr time series, a proxy for composition of mid-oceanic ridges and continental erosion, has power at long periods (>200 Myr) that increases towards the present day (see reddening in Fig. 3q). It also has high power between 490–380 Ma, 340–110 Ma, and during the last 40 Ma, at periods <50 Myr. The plate fragmentation time series also has greatest power at periods >200 Myr (Fig. 3t). However, nearly all power is concentrated at periods >50 Myr and at periods >100 Myr for the last ∼140 Ma (Fig. 3s–u).

A benefit to using wavelet transforms is that we can calculate the cross wavelet spectrum to objectively identify times and frequencies at which signals are large and similar. We first return to the problem of preservation, sampling, and the apparent similarity of rock package numbers, sea level, and biodiversity.

### Comparison of spectra

**Figure 4:**
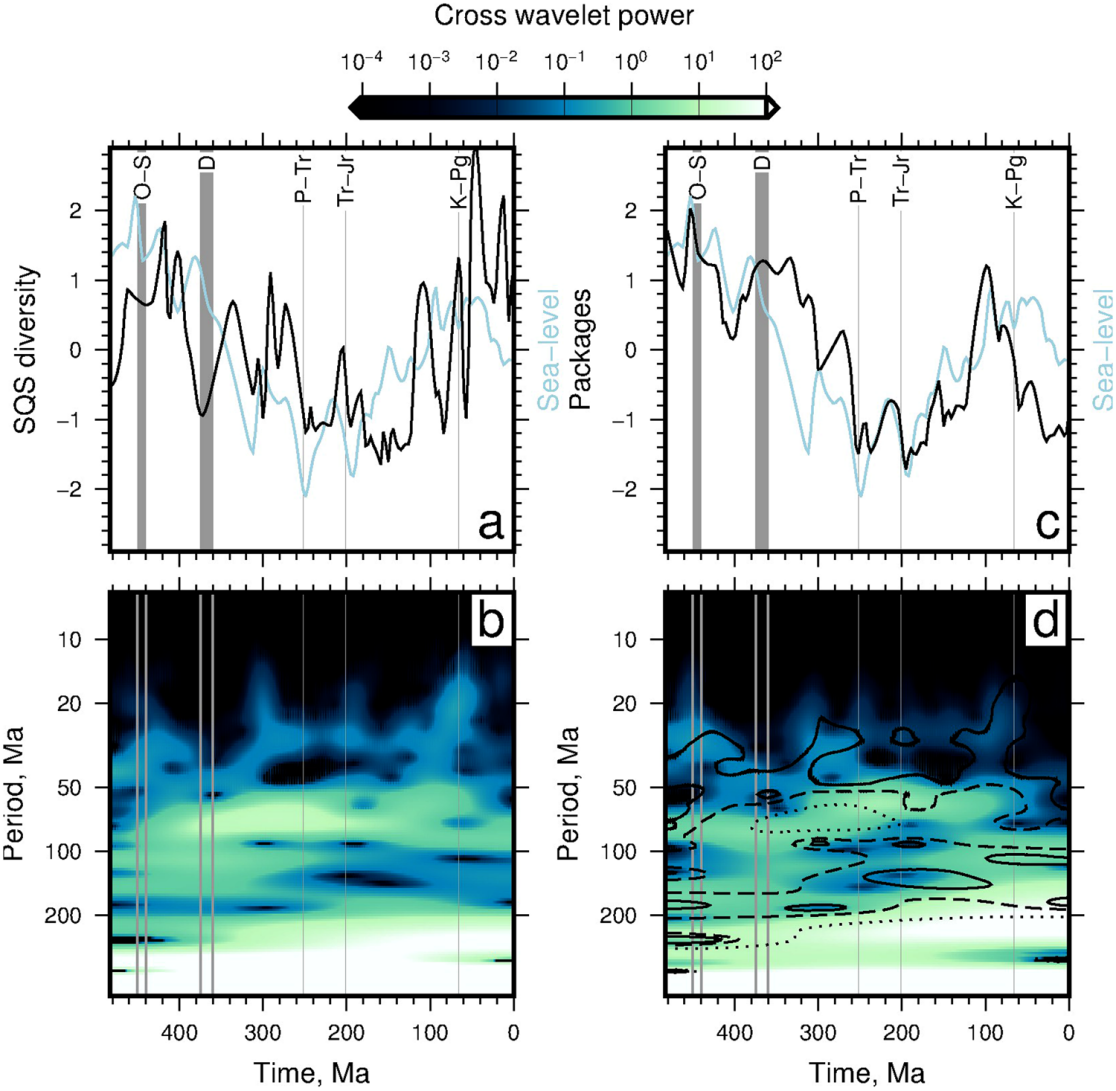
Comparison of the time-frequency content of SQS diversity, number of marine sedimentary packages and sea level time series. (**a**) Blue = sea level time series; black = SQS diversity. Gray labeled bands = mass extinction events. (**b**) Cross wavelet power spectrum of sea level and SQS diversity. (**c**) Blue = sea level; black = sedimentary packages. (**d**) Coloured contours = cross wavelet power spectrum for sea level and packages. Black contours = cross wavelet power spectrum from sea level and diversity (panel b): solid line = 0.1, dashed line = 1, dotted line = 10.

Figure 4 shows cross wavelet transforms for SQS diversity and sea level (panels a and b), and number of rock packages and sea level (panels c and d). Cross wavelet power is highest at periods >200 Myr and localised high power occurs at periods <200 Myr in both comparisons. Cross wavelet power is particularly high at periods of 50–100 Myr between times 400–200 Ma. At periods <50 Myr the SQS–sea level cross wavelet power is highest at times <400 Ma, and centred on ∼300 Ma, ∼200 Ma and ∼65 Ma. The similarity of the two cross wavelet power spectra (i.e. SQS–sea level and packages–sea level) is most apparent at periods ≳20 Myr (see contours in Fig. 4d).

**Figure 5:**
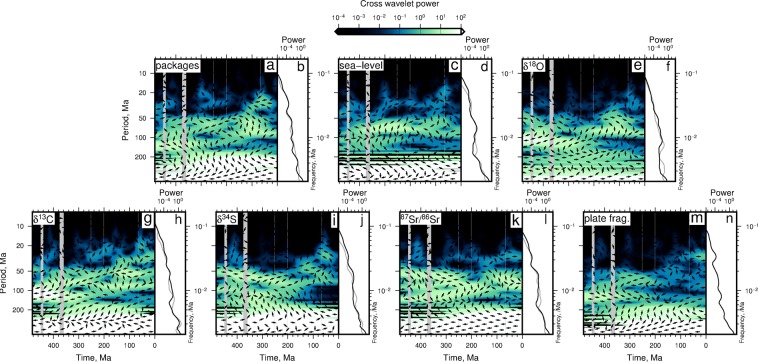
(**a**,**c**,**e**,**g**,**i**,**k**,**m**) Cross-wavelet power spectra of SQS diversity (*x*_*n*_) and labeled time-series (*y*_*n*_) resampled at SQS timescale (see^6^). Left pointing arrows = in phase. Right pointing arrows = out of phase by *π* (i.e. anti-phase). Up pointing arrows = phase difference of −3*π*/2. Down point arrows = out of phase by −*π*/2. Gray bands = mass extinction events. (**b**,**d**,**f**,**h**,**j**,**l**,**n**) Black = time-averaged cross wavelet power spectra. Gray = time-averaged cross wavelet spectra for genera and environmental variables (see Supplementary Fig. 6).

Figure 5 shows cross spectral power calculated using SQS diversity and environmental/geophysical variables. We also show calculated phase difference between each time series and the SQS diversity curve. Left pointing arrows show in-phase portions of the signals. Right pointing arrows indicate anti-phase signals (i.e. out of phase by *π*). Up and down pointing arrows show when phases lag by −3*π*/2 or −*π*/2, respectively (Supplementary Information^32^).

Figure 5a shows the cross wavelet power between SQS diversity and the number of rock packages, and their phase difference. The highest cross wavelet power (i.e. the parts of the two signals with high amplitudes at similar times and frequencies) is at periods >200 Myr (see white band at bottom of Fig. 5a). At the longest periods (≳200 Myr) these two signals are in phase (see also Fig. 1). There is also high power at periods of 100–200 Myr, between times ∼480–340 Ma, when the signals are anti-phase. Signals are also similar between periods of 50–100 Myr, and especially at ∼100 Ma. At shorter periods the signals are most similar between 100–0 Ma, when they are also in phase. The time averaged cross wavelet spectra is shown in Fig. 5b for the SQS time series (black curve) and, for comparison, the number of genera time series (grey curve) is also shown. The cross wavelet power for the SQS and raw genera curves are broadly similar and show that the similarity between the number of rock packages and biodiversity time series is greatest at longest periods. The peaks in the time averaged cross wavelet power indicate that there are up to 4–5 common periods of high power at >400 Myr, ∼200–300 Myr, ∼100–200 Myr, and centred on ∼50 Myr and ∼30 Myr.

The cross wavelet power between sea level and the SQS diversity curve is shown in Fig. 5c and its associated time averaged cross power is shown in Fig. 5d. These results show that greatest similarity between signals is at long periods (>300 Myr), where they are in phase. There is also a pronounced peak in time averaged power at periods ∼50–100 Myr, which is apparent for cross spectra calculated using either the SQS or raw genera time series. This peak appears to have been generated mostly between 380–200 Ma, during which there is a phase lag of ∼−*π*/2 (see down pointing arrows in Fig. 5c). There are at least four intervals of high power at periods <50 Myr at ∼440–420 Ma, ∼320–300 Ma, ∼200–190 Ma and ∼65 Ma, when the phase difference does not appear to have a simple pattern.

Figure 5e and f show cross wavelet power between the SQS and *δ*^18^O time series. Highest power is again at longest periods. However, the two signals appear to be anti-phase at these periods. They also appear to be anti-phase at periods of ∼200 Myr. At periods of ∼80–150 Myr, at times <350 Ma the two signals have high cross wavelet power and appear to be broadly in phase (see left pointing arrows in Fig. 5e). There is also considerable cross wavelet power between ∼200–100 Ma at periods of ∼50–100 Myr, which generates a broad peak in the time averaged spectra (Fig. 5f). There are at least four times of relatively high cross wavelet power at periods <50 Myr centred on ∼400 Ma, ∼300 Ma, ∼200 Ma and ∼65 Ma. The cross wavelet spectrum of SQS diversity and *δ*^13^C is broadly similar to that for *δ*^18^O (Fig. 5g–h).

Figure 5i shows the cross wavelet power of the SQS and *δ*^34^S time series. These time series have highest cross power at longest periods (>300 Myr) when they are in phase. Power is high for all times at periods >200 Myr. The time averaged spectra indicate that periods centred on ∼50 Myr have high cross wavelet power. At these periods the signals are broadly anti-phase. At periods <50 Myr highest power is most pronounced at two times: between 440–380 Ma and ∼60 Ma. Figures 5k and 5l show that the ^87^Sr/^86^Sr time series has broadly similar cross wavelet spectra to the *δ*^34^S time series. However, there are two important differences: first the cross wavelet spectrum at periods >200 Myr is in phase and, secondly, the ^87^Sr/^86^Sr-SQS cross spectra has additional peaks of relatively high power centred on ∼300 Ma and ∼200 Ma at periods <50 Myr.

The SQS and plate fragmentation cross wavelet spectra are shown in Fig. 5m,n. These signals are in phase at periods ≳300 Myr, when cross power is greatest. They are also in phase at periods ∼50–100 Myr between times 350–200 Ma, and at periods centred on ∼30 Myr at times <60 Ma, where there is a pronounced peak in the time averaged spectra. There are six times at which power is relatively high at periods <20 Myr: at times centred on ∼420 Ma, ∼300 Ma, ∼200 Ma, ∼120 Ma, ∼60 Ma and ∼10 Ma (see blue colouring in Fig. 5m).

In summary, cross wavelet power is highest at periods >200 Myr for all time series. High power is localised between periods of 50–200 Myr. Each time series has short lived (<100 Myr) intervals with pronounced short period (*T* < 50 Myr) cross wavelet power. These intervals often coincide with four of the five mass extinction events. SQS diversity and most environmental variables have higher cross wavelet power than number of raw genera at frequencies of ∼6 × 10^−3^ /Myr (*T* ∼ 170 Myr). Elsewhere the cross wavelet spectra of SQS diversity and environmental variables is similar to that for raw genera. Marine biodiversity is in phase with the number of marine sedimentary packages, sea level, strontium ratios, and plate fragmentation at periods >300 Myr. It is anti-phase with *δ*^18^O, *δ*^13^C and *δ*^34^S at these long periods. At periods of ∼100 to 300 Myr, raw genera and packages are out of phase by −*π*/2, and cross spectral power between raw genera and sea level is low. The raw genera time series is broadly anti-phase with *δ*^18^O, out of phase by −*π*/2 with *δ*^13^C, and by −3*π*/2 with *δ*^34^S. Shorter period phase differences can be seen in Fig. 5.

## Discussion

### Rock record and biodiversity

Similarities between both raw and SQS diversity time series and the sedimentary rock record are well-known (e.g.^6,17,34^). Our results suggest that similarities between marine biodiversity and sea level are generated at long (>200 Myr) and short (≲50 Myr) periods. One explanation, for example, is that the number of genera recorded is simply a function of the amount of rock sampled. An alternative explanation is that formation of sedimentary rock and biodiversity are driven by the same process or processes (e.g.^6^). For example, it seems reasonable to assume that increasing sea level will lead to both greater marine biodiversity and increased deposition of marine sedimentary rock. One way to test the latter hypothesis is to compare calculated power spectra of sea level, number of genera and rock packages. Clearly these time series must be drawn from independent datasets. The wavelet power spectrum of these three independent time series is broadly similar: high power at long periods; three intervals of high power at periods <50 Myr; time-averaged spectra similar to Brownian (red) noise (Figs 3 and 4). A simple interpretation of these results is that eustasy influences biodiversity, deposition of marine sedimentary rock, and its potential to preserve fossils (i.e. the quality of this record; e.g.^35,36^). Its influence is greatest at periods >200 Myr and at shorter periods (<50 Myr) for finite durations of time.

### Duration and periodicity of extinctions

The ‘Big Five’ Phanerozoic mass extinctions were first recognised by Raup & Sepkoski^37^, based on a compilation of the numbers of marine animal families through time (see also^38^). These mass extinction events were also identified in subsequent studies at the genus and species level^39^, as well as in the terrestrial realm^40^, and have generally continued to be recovered following the application of sampling standardisation techniques (e.g.^19^). However, whereas the most recent three mass extinctions (end-Permian, 252 Ma; end-Triassic, 201 Ma; end-Cretaceous, 66 Ma) all appear to have been geologically instantaneous (e.g.^41–43^), growing evidence indicates that the two earlier extinctions (Late Ordovician, and Late Devonian) were not discrete events. Instead, the Late Ordovician ‘event’ comprised two separate pulses of extinction between ∼446–443 Ma^44,45^, whilst the Late Devonian ‘extinction’ represents a long-term biotic crisis from ∼372–359 Ma, that was driven by anomalously low speciation rates^46,47^. This difference in duration is reflected in our spectral results, whereby the power at shortest periods (<10 Myr) is higher for the three most recent events than it is for the earlier extinctions (see dark blue colouring in Fig. 2e). We note that after the first two mass extinctions power remains low for ∼60 Myr, whereas after the last three events power (i.e. biodiversity) increases more rapidly. These observations support the view that the last three mass extinctions were geologically near instantaneous, whereas the first two were longer lived ‘events’.

Raup & Sepkoski^48,49^ identified a 26 Myr periodicity in the extinction rate of marine organisms during the Paleozoic. This was subsequently revised to a 27 Myr cycle, and extended to the entirety of the Phanerozoic by later authors^50,51^. Rohde & Muller^50^ documented an additional 62 ± 3 Myr periodicity in Phanerozoic marine animal biodiversity trends (see also^51–53^). These periodicities have been tied to an array of astronomical drivers (see^54^ for a summary), but have also been heavily criticised, with several authors arguing that there is no common cause driving extinctions over the last 500 million years (e.g.^38,46^). Most recently, *Erlykin et al*.^54,55^ argued that these periodicities are not statistically significant (see^53^ for a contrasting view). Many of these approaches make use of Fourier Transforms, which assumes signal stationarity. Our alternative methodology is to use wavelets to transform the time series, which does not assume that signals are stationary and avoids some of the spectra leakage associated with Fourier transformation.

Calculated time averaged spectra for the number of genera and SQS diversity time series contain peaks at approximately 27–31 Myr and 62–77 Myr, and also centred on ∼125 Myr and ∼333 Myr (see Fig. 2c and f;^23^). However, calculated spectra indicate that the spectral peak in the averaged time series are generated at finite intervals of time (Fig. 2). For example, the ∼27 Myr peak appears to have been generated principally during the last ∼200 Ma (see Fig. 2e). The recovery of a 27 Myr periodicity in biodiversity trends is novel to our study; previous evidence for periodicity on this time scale has been restricted to analyses of extinction rates. The broad double peak centered on ∼62 Myr appears to be generated by high power at slightly shorter periods (∼50 Myr) between ∼440–250 Ma, and at slightly longer periods (∼62 Myr) between ∼250–0 Ma (see Fig. 2e). The 125 Myr peak appears to be generated between ∼480–200 Ma, but diminishes in power towards the present. In summary, these time series do appear to contain periodicities, but they are of finite duration, as also supported by *Prokoph et al*.^23^’s wavelet analyses.

### What drives biodiversity?

One important observation is that most spectral power for the biodiversity time series resides at long periods (≳200 Myr). At these periods biodiversity is in phase with plate fragmentation, as well as sea level and ^87^Sr/^86^Sr time series. In some ways this apparent simplicity is unsurprising given that plate fragmentation probably drives sea level change and continental erosion. As such our results are in agreement with *Zaffos et al*.^9^ who suggested that plate fragmentation plays the fundamental role in driving long term changes in biodiversity.

Our results are also consistent with *Hannisdal & Peters*^6^’s conclusion that *δ*^34^S and sea level have strong ‘conditional information transfer’ to marine biodiversity. We can go further and identify times and timescales (frequencies) when these signals are similar or dissimilar, as well as the direction of ‘information flow’ through time (*sensu*^6^), using cross wavelet spectra.

Overprinted on these long term trends are shorter period changes in biodiversity and environmental variables. Some of these shorter term drivers are coincident with mass extinction events (e.g. Figs 3 and 4). We do find some support for short-term (∼27 Myr) periodicity, but this observation is for biodiversity trends, rather than solely extinction rates. Furthermore, our results indicate that this periodicity is restricted to the last ∼200 Myr, and did not characterise the entire Phanerozoic. It is unclear what might be driving this apparent periodicity and we agree with *Erlykin et al*.^54,55^ that there is currently no obvious mechanism for generating this signal. Instead, we suggest that short term changes in biodiversity are more likely driven by geological processes that have considerable variability at short time scales (e.g. glacio-eustasy, continental uplift and erosion, volcanism and asteroid impact). An additional complexity is the contribution of coevolutionary processes to biodiversity, which probably exert greatest influence at periods shorter than those considered herein (e.g.^12^).

## Conclusion

We show how continuous wavelet transforms can be used to identify where spectral power resides in biotic and environmental time series. Cross wavelet power spectra are used to identify where these signals are similar in time-frequency space. Our results have a number of implications for understanding the interplay of biodiversity and environmental changes on macroevolutionary scales. First, most spectral power in North American biodiversity time series is at periods ≳200 Myr, which we interpret as being primarily driven by long term plate fragmentation. Secondly, the biodiversity time series appears to be broadly consistent with a red noise spectrum (i.e. power is inversely proportional to frequency squared). The transformed time series indicate that periodicities of finite duration exist. For example, the one at ∼27 Myr appears to be restricted to the last ∼200 Myr. Finally, our results support the view that the first two Phanerozoic mass extinctions were longer-lived intervals whereas the most recent three events were geologically instantaneous. Transformation of, for example, global biodiversity and extinction rate time series, which are being rapidly updated, are avenues for future work and would test the generality of these results^56^.

## Supplementary information

Supplementary Information
