# Supplementary material for: Timing and periodicity of Phanerozoic marine biodiversity and environmental change
Source: Sci Rep. 2019 Apr 16;9:6116. doi: 10.1038/s41598-019-42538-7 (PMC6467882; doi:10.1038/s41598-019-42538-7)
Supplement: Supplementary file 1 — Supplementary Information [file 41598_2019_42538_MOESM1_ESM.pdf]

# Supplementary Material for Timing and Periodicity of Phanerozoic Marine Biodiversity and Environmental Change

Gareth G. Roberts & Philip D. Mannion

## 1. Data

The time series transformed were extracted from Hannisdal and Peters (2011) Supplementary Information. The following marine genera were included in their synthesis (and our analyses), Marine vertebrates: *Cetacea, Pinnipedimorpha, Pinnipedia, Otariidae, Phocoidea, Phocidae, Otarioidea, Odobenidae, Enhydra, Sirenia, Chelonioidea, Cheloniidae, Dermochelyidae, Protostegidae, Toxochelyidae, Thalassemyidae, Plesiosauria, Sauropterygia, Mosasauridae, Ichthyosauria, Thalattosuchia, Enaliosauria, Nothosauroidea, Placodontia, Thalattosauria.* Marine invertebrates: *Brachiopoda, Echinodermata, Cnidaria, Bryozoa, Porifera, Archaeocyatha, Hemichordata, Annelida, Priapulida, Granuloreticulosea, Actinopoda, Haptophyta, Bacillariophyta, Rhodophyta, Chlorophyta, Cyanobacteria, Rhizopodea, Bacillariophyceae, Pachycormiformes, Conodontophorida, Pleuronectiformes, Cephalopoda, Polyplacophora, Calpionellida, Aplacophora, Helcionelloida, Monoplacophora, Rostroconchia, Scaphopoda, Conodonta, Tunicata, Trilobita, Merostomata, Mollusca, Elasmobranchii.* We transformed their raw genera and SQS time series and environmental variables, which were calibrated using the 2004 Geological Time Scale. We note that their SQS and genera time series have slightly different temporal resolutions, and that they are also non-uniformly sampled through time ($1.5 \leq \delta_t \leq 14.2$ Ma), which complicates interpretation. All time series were linearly interpolated (resampled) to have constant temporal sampling, such that $\delta_t = 3$ Ma. To examine the impact of increased data resolution and the updated (2012) Geological Time Scale for our conclusions we transformed marine genera extracted the Paleobiological Database on $9^{th}$ March 2019. The genera transformed were the same as those examined by Hannisdal and Peters. The updated time series was evenly resampled prior to transformation (e.g. $\delta_t = 3$ Ma; see Figure 1).

## 2. Calculating cross wavelet spectra

Figure 2 shows cross wavelet transforms and phase calculations for a suite of synthetic examples. Figure 2a-e shows transforms for simple sinusoidal time series and their cross wavelet spectrum. Panel a shows signal $x_n = 2\sin(2\pi\omega t)$, where frequency, $\omega = 0.01$ (period = 100), panel b shows its wavelet transform. Panels c-d shows a time series, $y_n$, which has the same frequency content but smaller amplitude than $x_n$. Panel e shows the cross wavelet spectra for $x_n$ and $y_n$. Highest cross wavelet power is centred on a period of 100 and left pointing arrows show that theses signals are in phase. Panel j shows cross wavelet spectra for one signal, $x_n$, with a frequency, $\omega$, and another signal, $y_n$, composed of $x_n$ plus an additional higher frequency. Note that highest cross wavelet power exists for similar parts of signals (i.e low frequency content). Panels k-o show signals that are out of phase by $\varphi = \pi$ and have

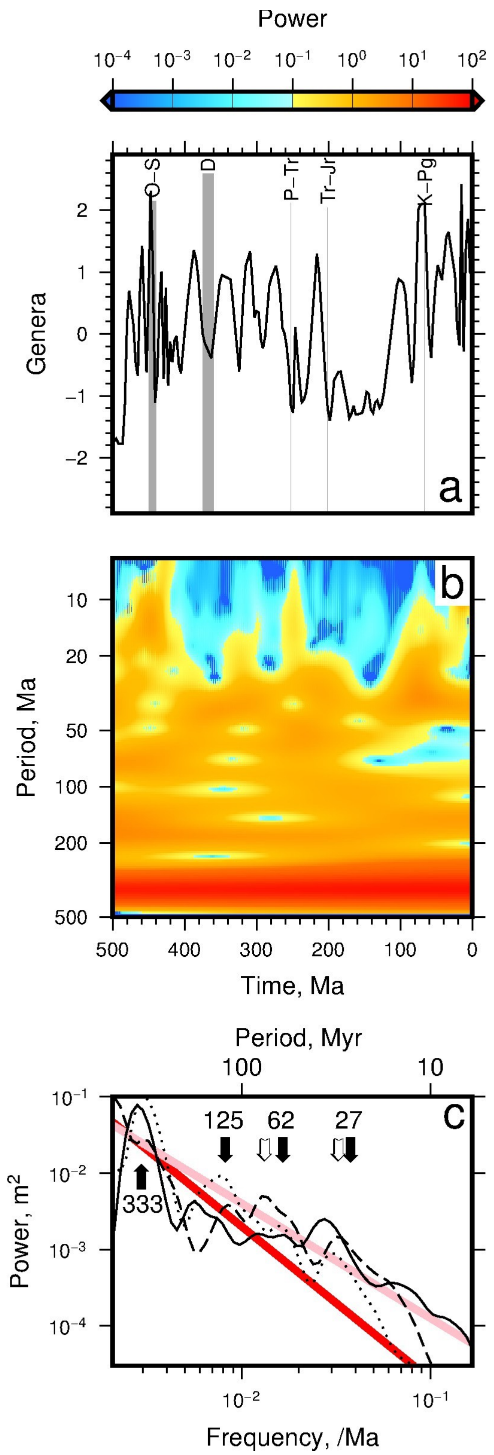


Figure 1: Power spectra of marine genera time series extracted from the PBDB on $9^{th}$ March 2019. (a) Genera time series. (b) Wavelet power spectrum. (c) Solid black curve = time averaged power spectrum for the updated time series. Dashed/dotted curves = time averaged SQS/raw genera power spectra (Figure 1c & 1f). Red/pink curves = red/pink noise (i.e. power proportional to $\omega^{-2}$ or $\omega^{-1.5}$. See Figure 1 for additional annotation.

different amplitudes, such that $x_n = 4\sin(2\pi\omega t + \varphi)$ and $y_n = 2\sin(2\pi\omega t)$. Note that arrows point to right for antiphase signals. Panels p-t show wavelet spectra for antiphase signals. Signal $x_n$ starts and stops, which leads to highest power in cross wavelet spectrum between times $\sim$ 150–350.

It is useful for our purposes to estimate phase difference between two time series. To make it easier to interpret our results we calculate cross wavelet power and phase angles for sinusoidal functions with known phases. In each example signal $y_n$ lags behind $x_n$ by phase, $\varphi$. Figure 3a shows signal, $x_n = 2\sin(2\pi\omega t)$ as a black curve and signal $y_n = 2\sin(2\pi\omega t + \varphi)$, where $\varphi = -\pi/4$. Panel shows cross wavelet spectrum and arrows indicate calculated phase. Panels (c)-(e) show results for signals that are $-\pi/2$ or $-3\pi/4$ out of phase. Panels g-l show cross wavelet spectra and phase for signals that are $-5\pi/4$, $-3\pi/2$ and $-7\pi/4$ out of phase. Note that it can be difficult to determine if signals lag or lead. For example, $y_n$ lagging $3\pi/2$ behind $x_n$ would give the same results as $x_n$ leading $y_n$ by $\pi/2$.

## 3. Spectra for Marine Invertebrate Genera

The impact of different sampling techniques on our results is assessed by transforming time series using the timescale from the genera dataset. Figure 4 shows spectra for the environmental variables sampled at the same times as the genera time series shown in Figure 2d. As expected calculated spectra is almost indistinguishable from the time series sampled using the SQS timescale. Cross wavelet spectra between sea level, number of rock packages and genera is shown in Figure 5. The largest difference between this cross wavelet spectra and that calculated using the SQS diversity curves is a localised increase in cross wavelet power between periods of 100–50 Myr between 320–200 Ma, and an increase in power between periods of 50–20 Myr during the Ordovician-Silurian. Otherwise calculate cross wavelet spectra are similar, which suggests that the comparisons between time series is reasonably stable. The cross wavelet spectra between genera and environmental variables are also vary similar to those sampled using the SQS timescale (Figure 6).

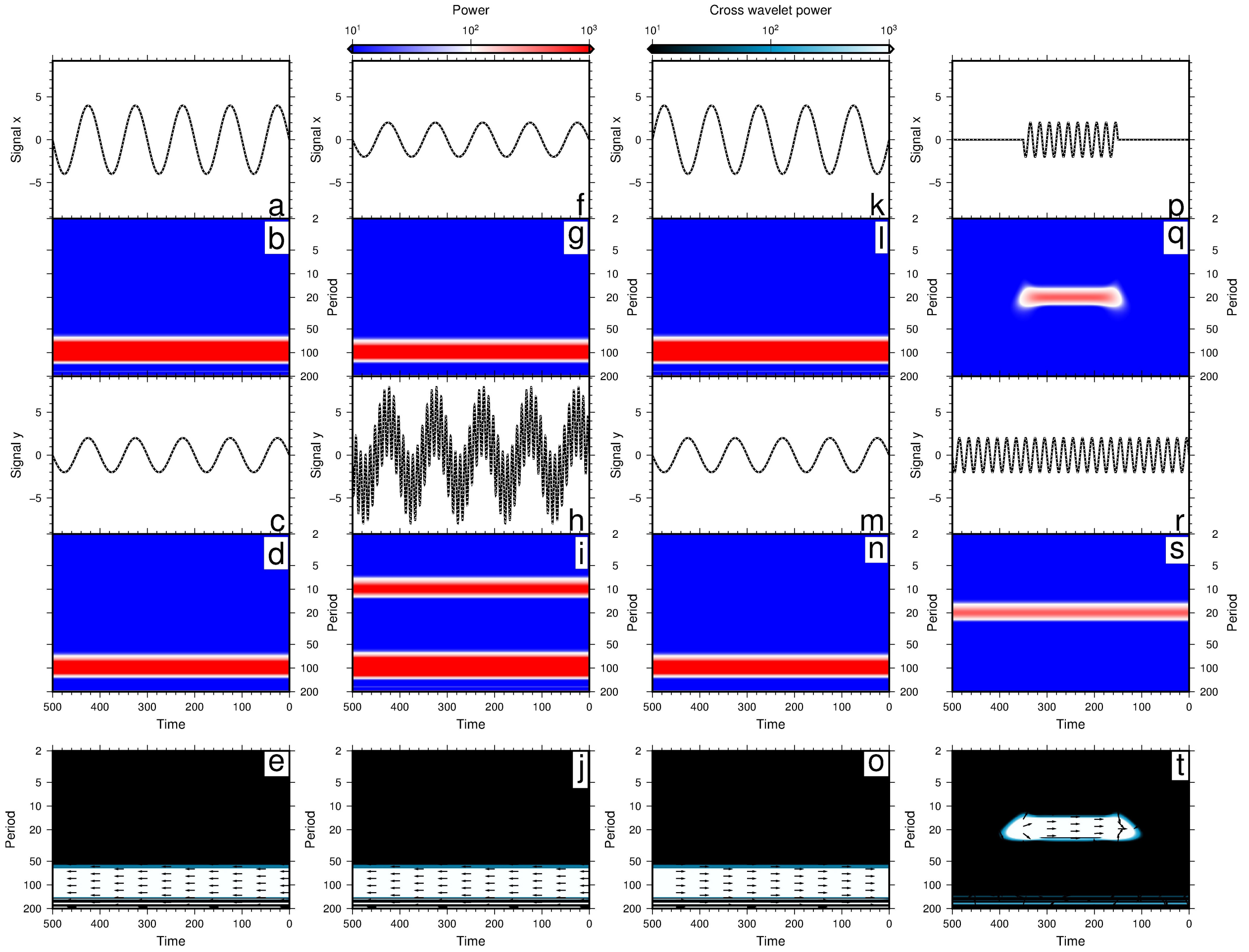


Figure 2: Cross wavelet power spectra for synthetic examples. (a) Signal $x_n = 4\sin(2\pi\omega t)$. (b) Wavelet power spectrum of $x_n$. (c-d) Signal, $y_n$ and its power spectrum. (e) Cross wavelet spectrum of signals $x_n$ and $y_n$. (f-j) Signals have different frequency content. (k-o) Antiphase signals with different amplitudes. (p-t) Antiphase signals with different time localisation. Left and right pointing arrows indicate in phase and antiphase signals, respectively.

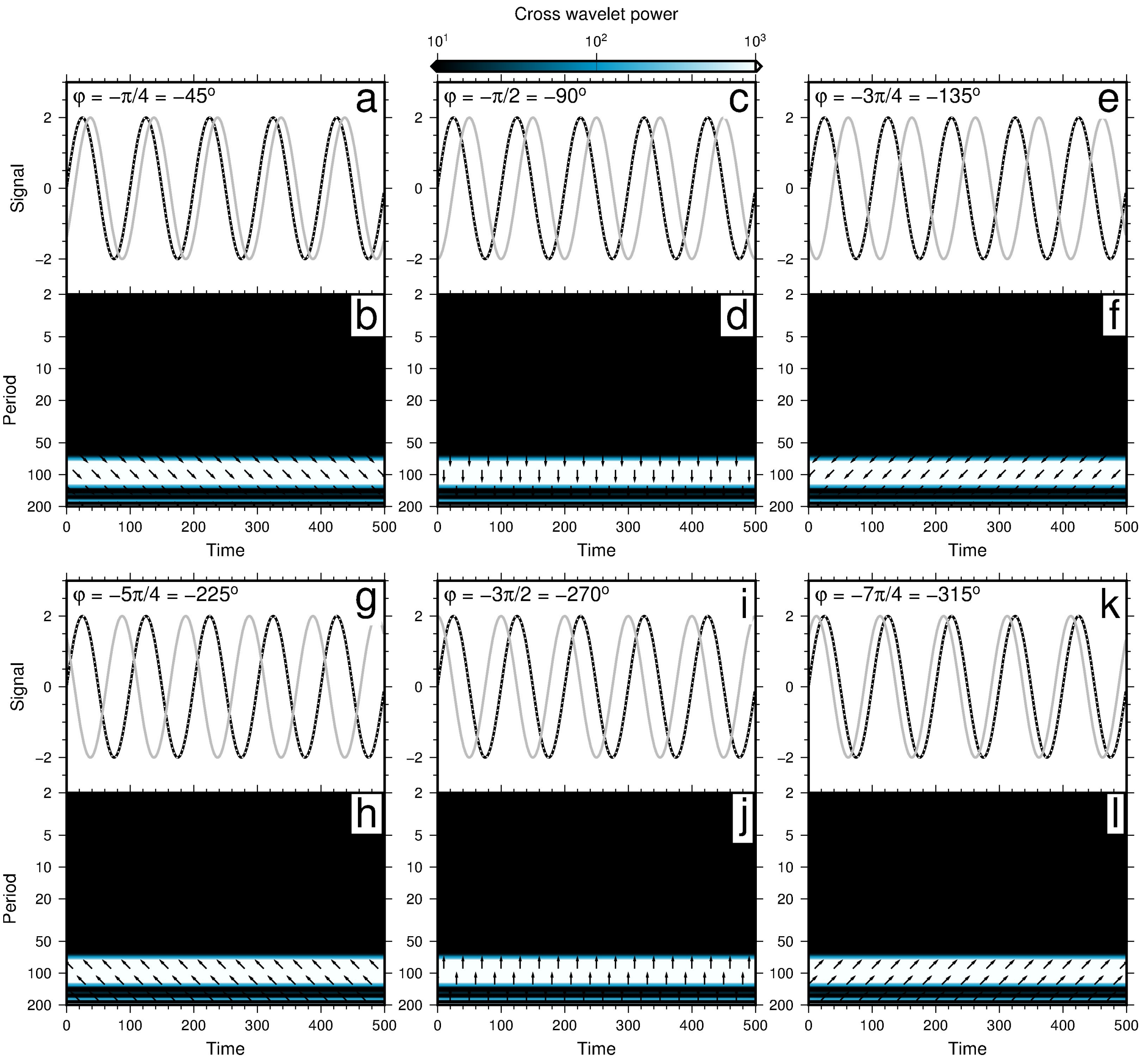


Figure 3: Cross wavelet spectrum and phase lag. In each example signal $y_n$ lags behind $x_n$ by phase, $\varphi$. (a) Black: signal $x_n = 2\sin(2\phi\omega t)$; grey: $y_n = 2\sin(2\pi\omega t + \varphi)$, where $\varphi = -\pi/4$. (b) Cross wavelet power of $x_n$ and $y_n$ and calculated phase angle (arrows). (c-d) Phase difference, $\varphi = -\pi/2$. (e-f) Phase difference, $\varphi = -3\pi/4$. (g-h) Phase difference, $\varphi = -5\pi/4$. (i-j) Phase difference, $\varphi = -3\pi/2$. (k-l) Phase difference, $\varphi = -7\pi/4$.

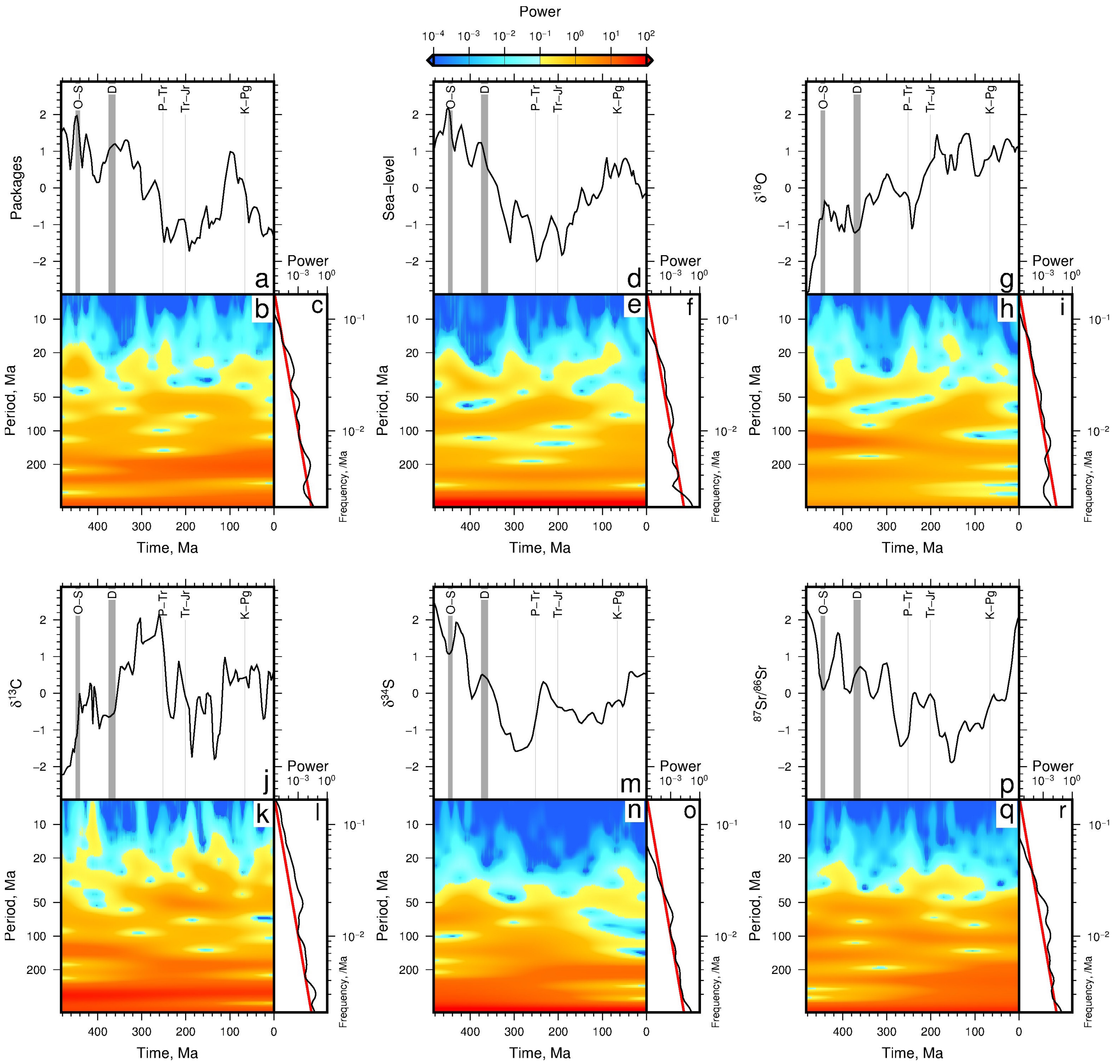


Figure 4: (a) Black line = time series of number of marine sedimentary packages from Hannisdal & Peters (2011). (b) Wavelet transform power spectrum of packages sampled using Hannisdal & Peters's (2011) genera time scale. Highest power is at long wavelengths. (c) Time-average power rectified by scale. Red line = red noise power spectrum. Wavelet transforms in (d-f) are for continental flooding, (g-i) $\delta^{18}$O, (j-l) $\delta^{13}$C, (m-o) $\delta^{34}$S, (p-r) $^{87}$Sr/$^{86}$Sr.

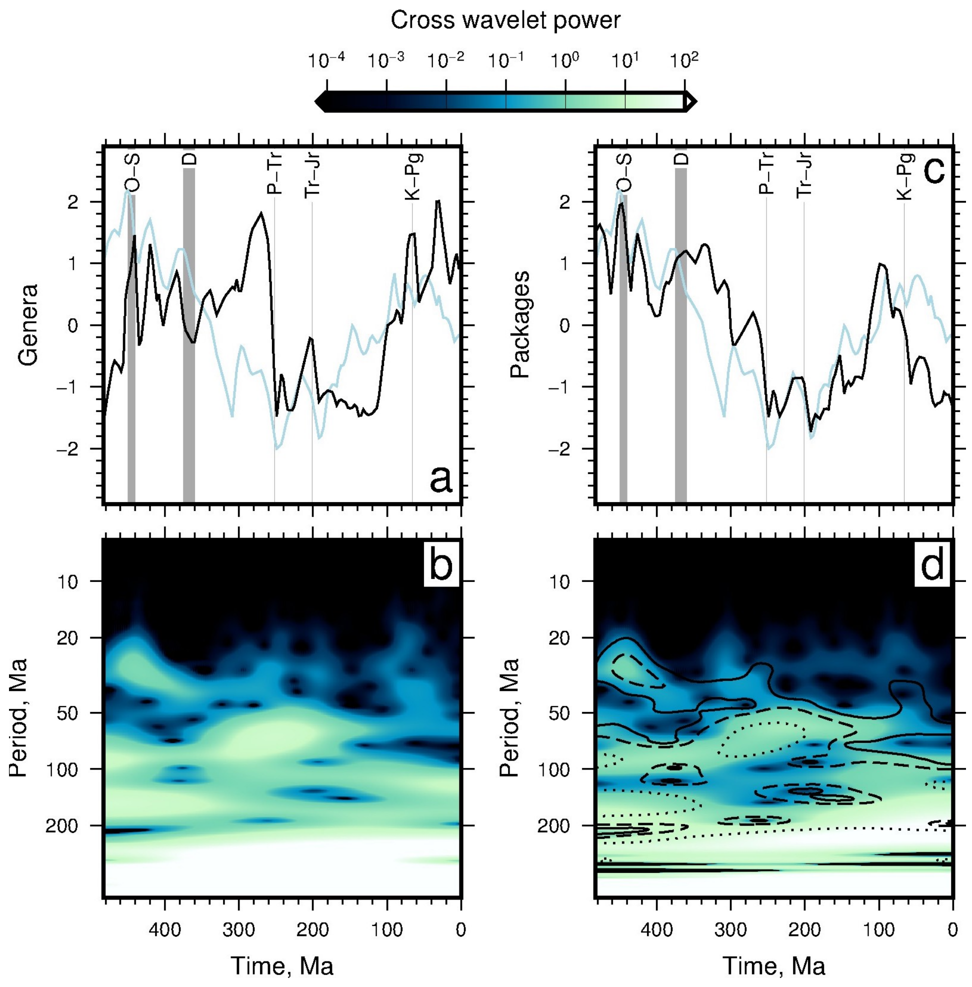


Figure 5: Cross wavelet power spectra. Comparison of time-frequency content of sea level, genera and marine sedimentary packages. (a) Blue = sea level time series; black = genera. Gray labeled bands = mass extinction events. (b) Cross wavelet power spectrum of sea level and genera. (c) Blue = seal level; black = sedimentary packages. (d) Coloured contours = cross wavelet power spectrum for sea level and packages. Black contours = cross wavelet power spectrum from sea level and genera (panel b): solid line = 0.1, dashed line = 1, dotted line = 10.

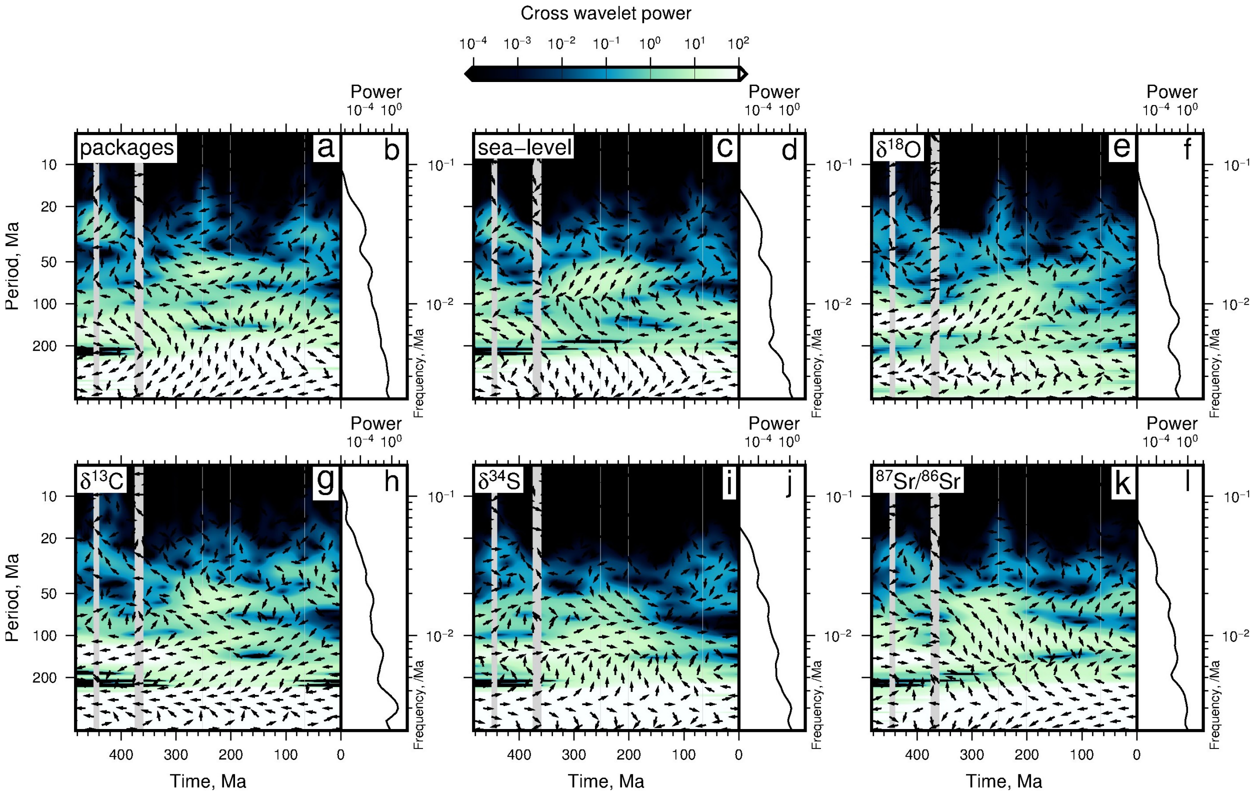


Figure 6: Cross-wavelet power spectra of genera ($x_n$) and labeled time-series ($y_n$). High cross wavelet power indicates that wavelengths and amplitudes of time series are similar in frequency-time space. Arrows indicate relative phase. Left pointing arrows = in phase. Right pointing arrows = anti phase. Up pointing arrows = phase difference of $-3\pi/2$. Down point arrows = out of phase by $-\pi/2$. See Supplementary Figure 2. Gray bands = mass extinction events. (b, d, f, h, j, l) Time-averaged cross wavelet power spectra.
